# Arthroscopic lateral collateral ligament imbrication for the treatment of posterolateral rotatory elbow instability

**DOI:** 10.1016/j.jseint.2024.09.024

**Published:** 2024-10-16

**Authors:** Christos Koukos, Nikolaos Platon Sachinis, Konstantinos Sidiropoulos, Michael Kotsapas, Kerem Bilsel, Fredy Montoya

**Affiliations:** a Sports, Trauma and Pain Institute, Thessaloniki, Greece; b First Orthopaedic Department of Aristotle University of Thessaloniki, “Georgios Papanikolaou” Hospital, Thessaloniki, Greece; c Emergency Department, Medical School of Patras, University of Patras, Greece/Papageorgiou General Hospital of Thessaloniki, Thessaloniki, Greece; d Acibadem University, Fulya Acibadem Hospital, Instabul, Turkey; e Universidad de Concepcion, Sanatorio Aleman Clinic, Concepcion, Chile

**Keywords:** Elbow, Arthroscopy, PLRI, Imbrication, LUCL, Elbow instability

## Abstract

**Hypothesis:**

Posterolateral rotatory instability (PLRI) of the elbow is commonly treated with open lateral collateral ligament (LCL) reconstruction techniques. This cohort study evaluates the efficacy of a less invasive arthroscopic LCL imbrication technique for reducing grade I or II PLRI.

**Methods:**

Forty-three patients with stage 1 or 2 PLRI, unresponsive to conservative therapy, were included. Diagnoses were based on chronic post-traumatic pain (11 patients), chronic atraumatic lateral elbow pain (20), and previous open tennis elbow surgery (12). Following clinical and arthroscopic diagnosis confirmation, the LCL imbrication technique was performed. The Mayo Elbow Performance Score and range of motion (ROM) were assessed preoperatively and postoperatively using the Shapiro-Wilk test and Wilcoxon signed rank test, respectively, with a minimum 12-month follow-up (range 12-48 months).

**Results:**

The Mayo Elbow Performance Score increased significantly from a median of 45 points preoperatively to 90 (range 80-100) at 3 months and 95 (range 80-100) at 12 months follow-up (*P* < .001). Postoperative median flexion reached 140°, and extension was 0°. At 12 months, 2 patients experienced a 10° extension deficit; 95.3% (41 of 43) achieved full ROM. Knot irritation occurred in 4 patients (out of the first 10 of this cohort, 9.3%), 3 of them requiring knot removal. Switching to a polydioxanone 1 suture eliminated this complication. One patient underwent arthroscopic arthrolysis for adhesions after 14 months.

**Conclusion:**

Arthroscopic LCL imbrication offers favorable outcomes for grade I or II PLRI from the third postoperative month with minimal complications. A slight restriction in ROM and transient knot discomfort were the main issues, the latter resolved by switching to a thinner polydioxanone suture.

First described by O'Driscoll et al in 1991, posterolateral rotatory instability (PLRI) of the elbow results from incompetence of the lateral ulnar collateral ligament (LUCL), which plays a critical role in resisting varus stress and stabilizing the radial head against posterior subluxation or dislocation.^9^ Recent studies, however, suggest a more extensive compromise of the lateral collateral ligamentous complex; the LUCL may be only contributing a portion to, rather than being the major constraint to PLRI, as part of the whole lateral collateral ligament complex with its surrounding tissues.^3^^,^^6^

Three stages of PLRI have been described.^9^ In stage 1, the elbow subluxates in a posterolateral rotatory direction, evidenced by a pivot-shift test. Stage 2 consists of elbow incompletely dislocated with the coronoid process perched under the trochlea. Stage 3 occurs when there is a full elbow dislocation, positioning the coronoid process behind the humerus. This stage can be subclassified into 3 categories: stage 3A, which has an intact anterior band of the medial collateral ligament (MCL), making the elbow stable to valgus stress after reduction; stage 3B, with a disrupted anterior band of the MCL, and an unstable elbow during valgus stress after reduction; stage 3C, completely unstable elbow due to the humerus being stripped of all soft tissue.

Treatment techniques for PLRI vary widely, with no consensus on the optimal approach. Commonly, open reconstruction of the LUCL using tendon grafts—such as the palmaris longus, semitendinosus, gracilis tendons, or triceps aponeurosis—is used. Minimally invasive options are available for the initial stages of PLRI. Arthroscopic interventions aim to achieve one or both of the following: plication of the complex’s major components or reattachment of the complex to the humerus.^11^ Arthroscopic LCL plication has been documented since 2001. Roger van Riet expanded this approach by incorporating the entire LCL complex, lateral capsule, and anconeus into a single doubled suture, effectively imbricating these structures.^7^ This cohort study aims to discuss the short-term outcomes, risks, and benefits of a modified elbow imbrication procedure.

## Methods

### Study design

A retrospective analysis was performed on prospectively collected data from 43 patients who underwent arthroscopic LCL imbrication between 2019 and 2023. These procedures were all carried out by the same surgeon in a sports injury department at a district hospital. Eligibility for the procedure was determined by symptoms of instability such as clicking, lateral-sided pain, apprehension during push-ups, or manifest instability; a positive result in at least 2 out of 3 clinical PLRI tests (pivot shift, posterior drawer, and table top tests); and symptom persistence for more than 6 weeks. Surgery required the presence of stage 1 PLRI (radial head subluxation) or stage 2 PLRI (coronoid perching on the humerus) during intraoperative testing. Exclusion criteria included no complete or extensive partial tear of the common extensor origin on preoperative magnetic resonance imaging, multidirectional instability, skeletal instability, open wounds or nerve damage, current infections, and medical unfitness.

### Preoperative evaluation

Patients underwent preoperative evaluations, including radiographs and magnetic resonance imaging scans. Assessments of elbow range of motion (ROM) using a goniometer and the Mayo Elbow Performance Score (MEPS) were conducted, with MEPS ranging from 5 (lowest score) to 100 (highest score) [6]. Follow-up visits were conducted by the surgeon and a fellow, with a minimum duration of 12 months (range 12-48 months), measuring outcomes at 3 months, 6 months, 12 months, and annually thereafter.

### Surgical technique

**Figure 1:**
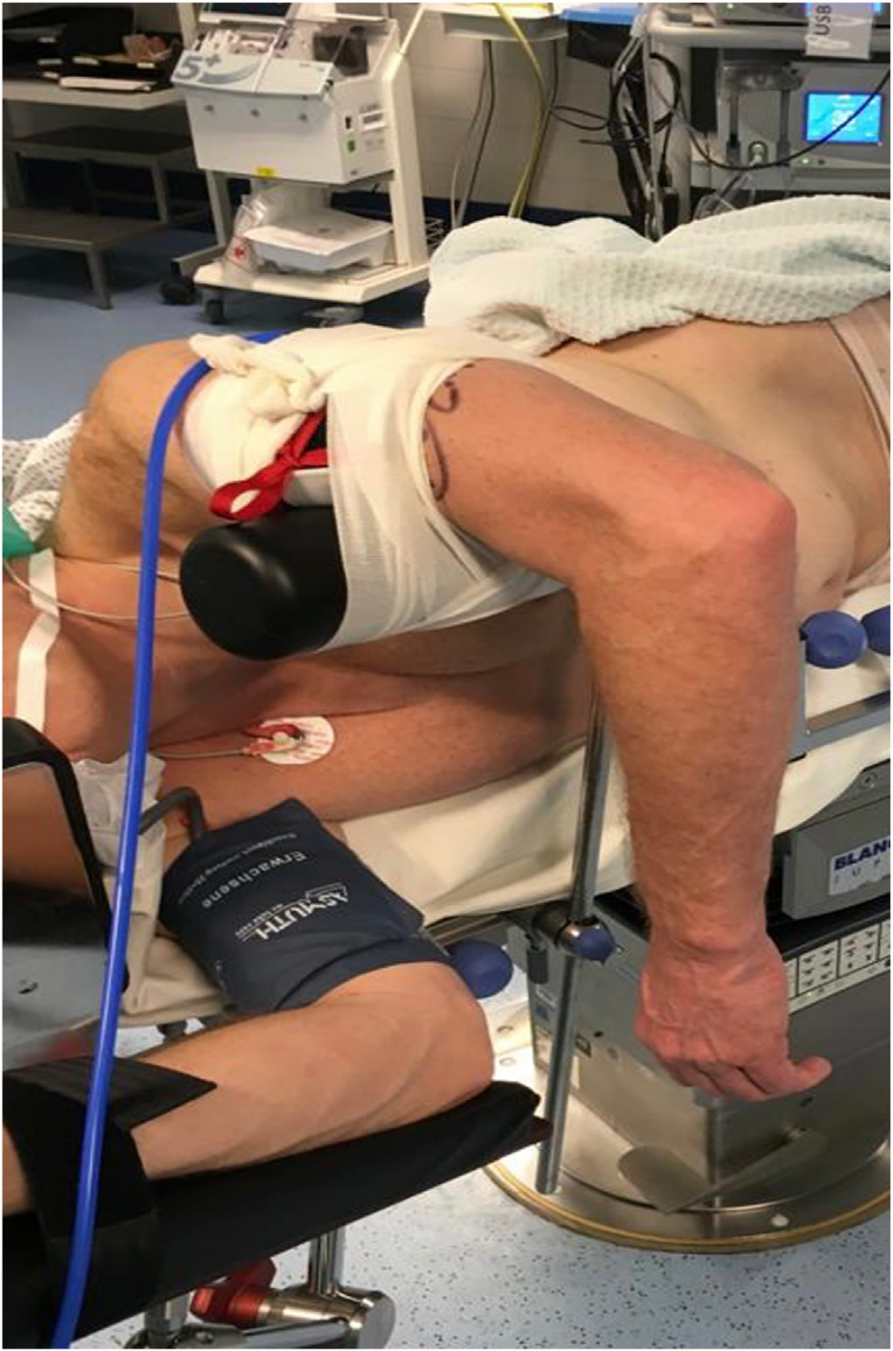
Positioning of patient in the lateral decubitus position.

The technique alone has been extensively described and has been recently been published. Under general anesthesia, the patient is first inspected. The pivot shift, forceps grip test, and posterior drawer tests are all performed and reported, as well as ROM and varus and valgus stress. The technique is then performed with the patient in a lateral decubitus position (Fig. 1). Applying varus stress to the elbow identifies the LCL complex and its insertion. If the anterior compartment of the joint is to be investigated, an anteromedial portal can be made before viewing the posterior sections.

The scope is inserted into the posterior compartment through a high posterolateral portal just lateral to the triceps, roughly at the same height as the olecranon tip. The scope is aimed first to the ulnar gutter. We assess medial stability by inserting a 4-mm trocar from a transtricipital portal between the medial side of the olecranon tip and the trochlea. If it does not open, the MCL is intact.

**Figure 2:**
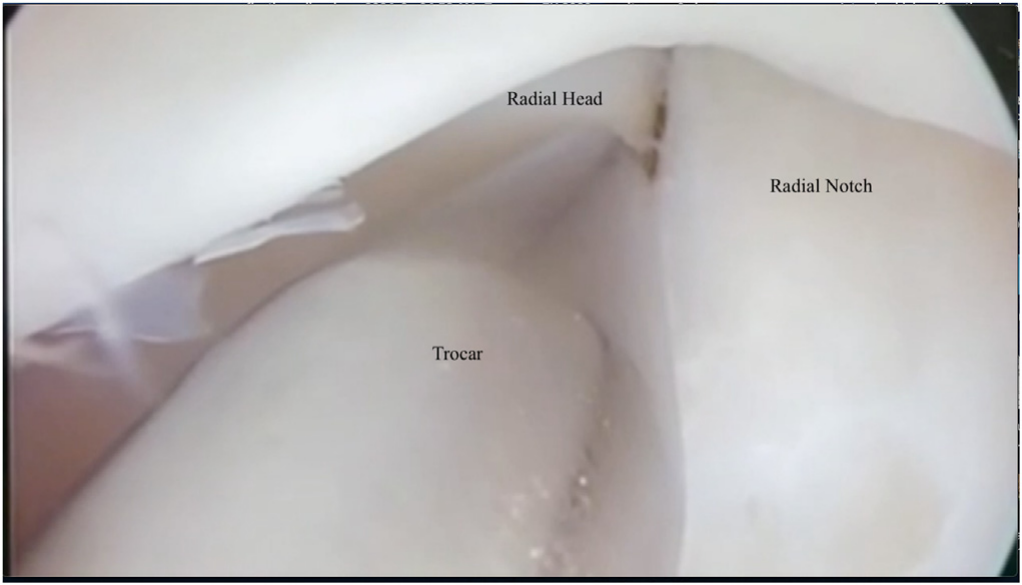
Arthroscopic elbow view from the posterolateral portal. Intraoperative testing for lateral collateral ligament instability.

The scope is then inserted into the radiohumeral gutter. A soft spot portal is established to remove any synovium that is impeding the vision. The Arthroscopic Rotatory Instability test, as well as the drive-through sign and trocar test performed from the posterior compartment, aid in the diagnosis of PLRI (Fig. 2) [7, 8]. Once the indication has been validated, we proceed with the procedure. A polydioxanone (PDS) I suture is threaded through a 14G needle (after the first 10 patients of this cohort, the lead surgeon switched to using a PDS I suture instead of a II, due to known irritation issues, as discussed in the results section). This is then inserted immediately adjacent to the lateral epicondyle. Under direct supervision, the needle is directed to the radiohumeral gutter, and the suture is shuttled into the joint. The suture is drawn through the soft spot portal with a grasper.

**Figure 3:**
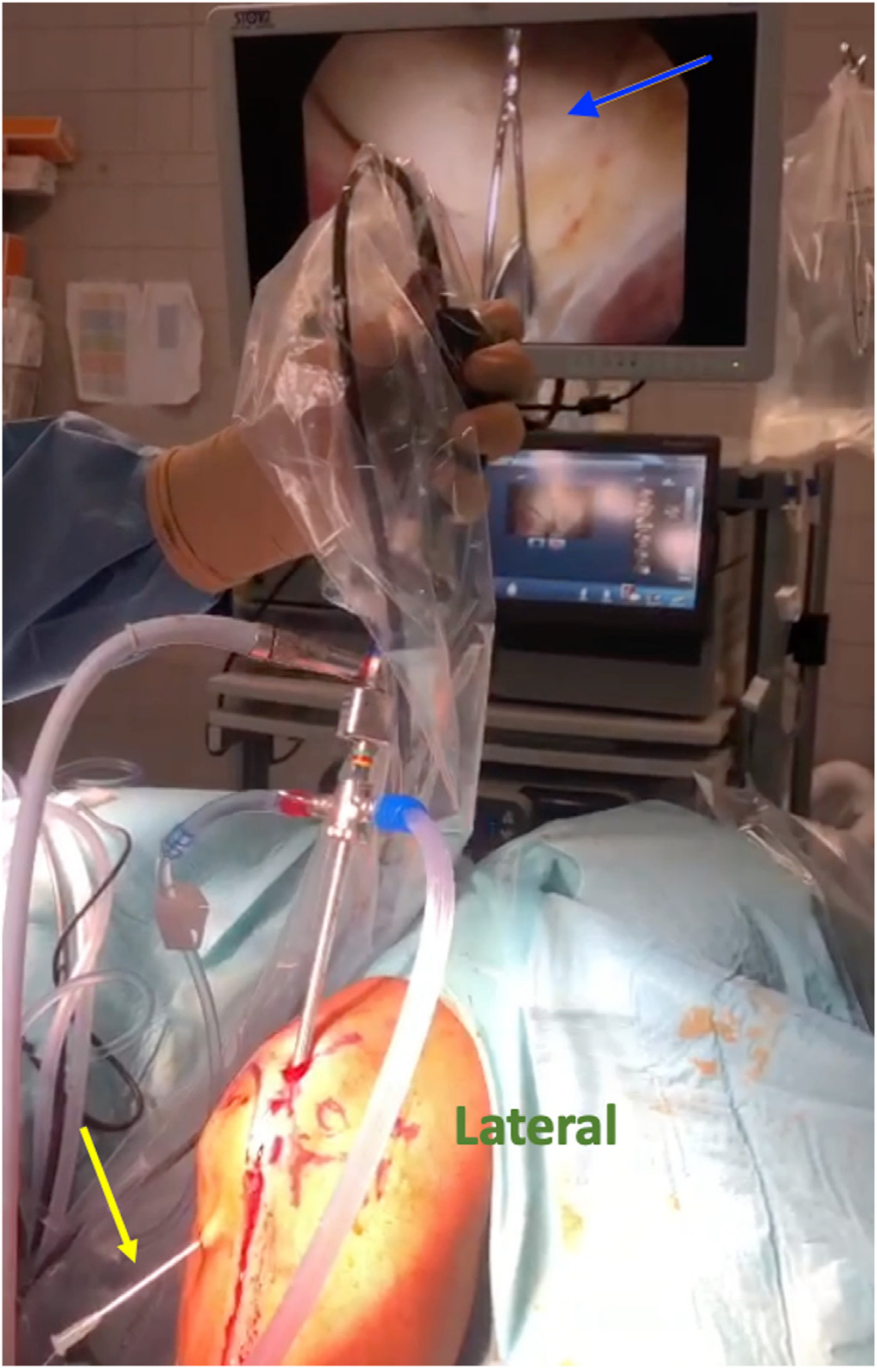
Passing of CHIA percpasser ( in monitor) through the needle ().

The 14G needle is then inserted directly on the bone posterior to the LUCL insertion and into the radiohumeral gutter, and a CHIA percpasser (DePuy Mitek, Raynham, MA, USA) is driven through the needle (Fig. 3). The CHIA is then dragged through the soft spot portal and knotted with the end of the PDS suture, which was entered from the lateral epicondyle in the same manner as the CHIA. The PDS is then pulled so that the knotted end of the CHIA exits the lateral epicondyle area. The knot is cut, and a looped PDS II suture is put onto the CHIA and drawn through the LUCL insertion site, allowing both ends of the looped PDS to escape that site. At this stage the loop of the PDS rests out of the lateral epicondyle center point and both of its ends are coming out of the LUCL insertion area.

**Figure 4:**
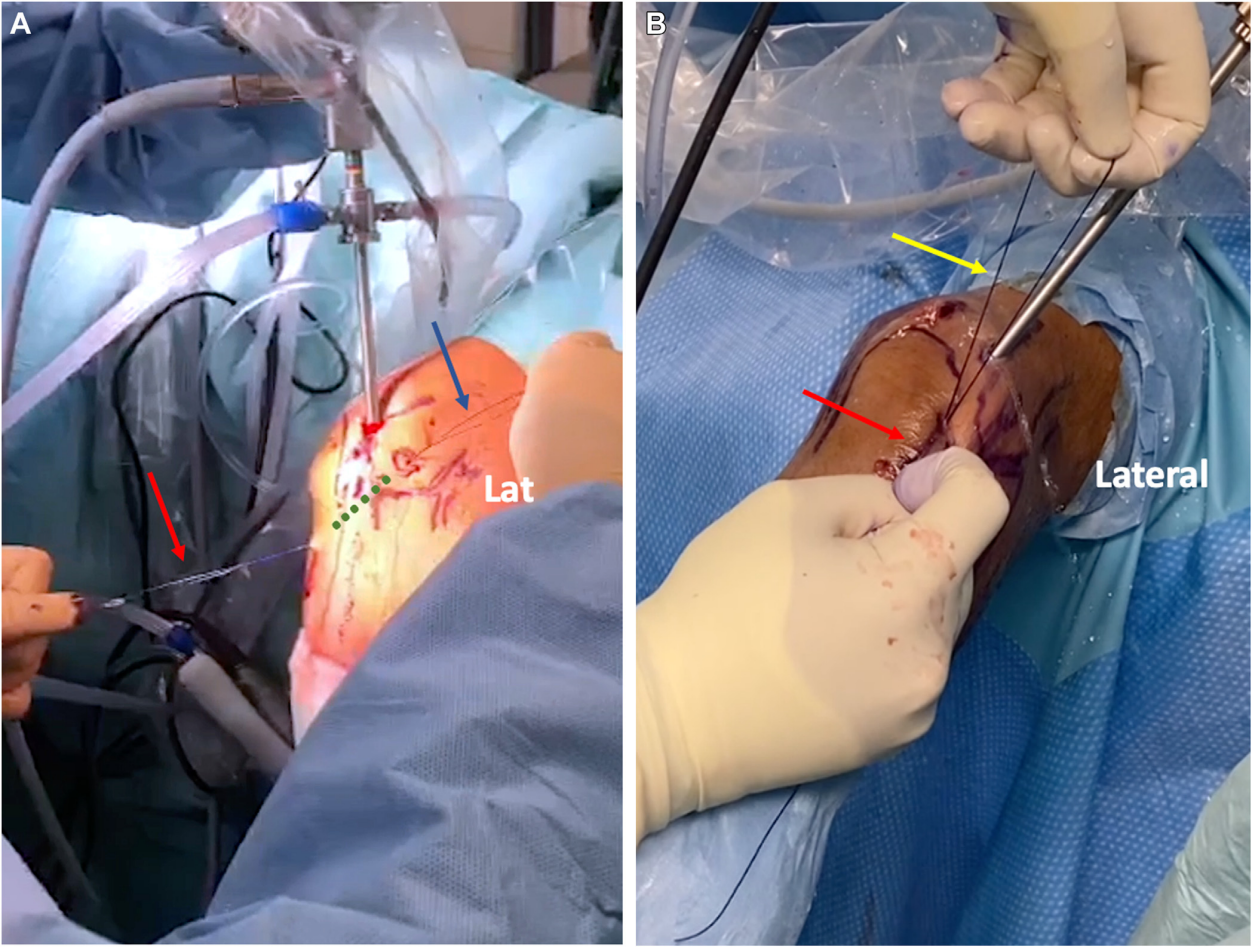
(**A**) Passing of PDS suture subcutaneously from proximal () and distal sites (). (**B**) Exit of the PDS suture through the soft spot portal (loop: , ends: ). *PDS*, polydioxanone.

Next, the looped portion and both ends of the PDS suture are retrieved subcutaneously through the soft spot portal with a tiny clamp (Fig. 4). With the elbow flexed at 60° and full pronation, the loop and ends are tightened. When both the drive-through sign and the Arthroscopic Rotatory Instability test are negative, proper tension has been established. The arthroscope is then drawn back, and a Nice knot is made to avoid several knots that cause irritation. The sutures tighten and fortify the lateral capsule, anconeus, and LCL complex in this manner, and scope reinsertion and probing examination may ultimately confirm the drive-through negative sign. Following that, all ports are closed with a 3-0 nylon suture and a posterior splint is put for comfort for the first 48 hours. The patient is then fitted with a dynamic brace for 6 weeks. Extension is permissible up to 30^o^ for 4 weeks. Full extension is permitted with the dynamic brace on beginning in the fifth postoperative week and supination is avoided for a total of 6 weeks.

### Statistics

Data are presented as medians with ranges in parentheses and were analyzed with the SPSS software (v.26; IBM Corp., Armonk, NY, USA). The Shapiro-Wilk normality test was used to determine the sample’s normal distribution. A Wilcoxon signed rank test was used to compare preoperative and postoperative MEPS scores. We used the Mann-Whitney test for clinical outcomes analysis, as normality assumptions failed. For subgroup analysis, we utilized the Kruskal-Wallis tests. A statistical significance was defined when *P* < .05.

## Results

This study included 43 patients (20 male, 23 female), with a median follow-up period of 30 months (range: 12-48 months). No participants were lost to follow-up. The median age was 48 years, ranging from 20 to 62. The predominant cause of PLRI was chronic elbow pain with no obvious previous history of trauma in 20 patients. For 11 patients, a traumatic event was established from their medical history, while a postsurgical cause (previous tennis elbow release) was identified in 12 patients. Among these, 28 presented with stage 1 PLRI and 15 with stage 2 PLRI. The median duration from symptom onset to surgical intervention was 12 months (range 6-48 months). Cointerventions during arthroscopic LCL imbrication included synovial tissue débridement in 36 patients and loose body removal in 4.

Preoperatively, the median ROM was 140° (range: 90°-140°). By the final follow-up, it was still 140°, with median extension reaching 0°, although this change was not statistically significant (*P* = .76). The MEPS significantly improved from a preoperative median of 45 (range 30-80) to 90 (range 80-100) at 3 months, increasing to 95 (range 95-100) at the 12-month follow-up (*P* < .001). Beyond 12 months, no further significant changes in ROM or MEPS were observed. All patients reported stable elbow function at their last assessment.

### Complications

By 12 months postoperation, an extension deficit of 10° was observed in 2 patients, equating to 95.3% (41 of 43) achieving full ROM. Knot irritation was observed in 4 patients (9.3%), all within the first 10 participants of the cohort. Three patients required knot removal under local anesthesia 6 months postoperation, one of them scoring 80 on MEPS. To mitigate this issue, the lead surgeon transitioned from using PDS II suture to PDS I suture, after which no subsequent cases of knot irritation were reported. Additionally, 1 patient required arthroscopic arthrolysis to address adhesions 14 months postoperation.

## Discussion

In our study, we evaluated 43 patients with stage 1 and 2 PLRI, employing a less invasive LUCL imbrication technique with a double strand of PDS suture. We observed significant clinical improvement as early as the first postoperative follow-up at 3 months, with all participants reporting stable elbow function sustained through to the final follow-up.

PLRI is mainly a consequence of trauma in 94% of patients, involving either an elbow dislocation or a fall with outstretched, supinated forearm.^1^^,^^5^^,^^9^ It may also develop as an iatrogenic complication of multiple corticosteroid injections or lateral epicondylitis surgery, as well as from cubitus varus due to the attenuation of ligaments.^8^ When the radial collateral ligament does not heal properly and is nonfunctional, the radial head rolls off of the capitellum during forearm supination. As the radial head subluxates posterolaterally, the ulnohumeral joint begins to gap, and as the pattern of instability continues the entire elbow joint may dislocate.^9^

Its clinical presentation may vary and its symptoms may have an insidious course, making its diagnosis challenging to the inexperienced physician. Patients often complain of discomfort or lateral-sided pain during activities that require extension and supination, associated often with locking or catching during extension of the elbow^4^^,^^9^^,^^10^[1,5,8]. Many patients with chronic PLRI present with a seemingly normal elbow, exhibiting full ROM and no palpation-induced pain [10].

While open surgical interventions for early-stage PLRI exist, arthroscopic techniques offer a minimally invasive alternative to restore stability by reattaching the lateral collateral ligament to the humeral insertion.^2^^,^^7^^,^^12^^,^^13^ Smith and colleagues were the first to perform arthroscopic LUCL plication as a minimally invasive surgery in 2001. Their method involves threading 4-7 separate absorbable sutures from the lateral ulnar border to the tissue posterior to the lateral epicondyle. After knot-tying, the LCL can then be efficiently plicated.^13^ Our method is simpler, arguably faster, and it reduces the number of subcutaneous knots, which could cause soft tissue irritation.

This technique demonstrated satisfactory outcomes for subacute and chronic stage 1 and 2 PLRI, aligning with findings from Savoie et al, which suggest that arthroscopic interventions may rival the efficacy of open surgeries.^12^ Our technique may not be as demanding as other plication techniques due to its lower number of sutures utilized, but it requires precision and a thorough understanding of elbow anatomy.

As a cohort study, it has several limitations related to its retrospective nature. A priori power analysis was not performed. Diagnosing and staging of PLRI may vary even among specialist surgeons.^14^ Patient selection bias may exist; however, we tried to limit the overtreatment tail of that bias by establishing correct diagnosis intraoperatively. Also, the surgeon and fellow examining patients during follow-up were not blinded to the procedure. It is also debatable whether the outcomes measured are appropriate for assessing an unstable elbow.^7^

## Conclusion

Our findings affirm the efficacy of arthroscopic LCL imbrication in treating grade I or II PLRI, achieving stable elbow function from the third postoperative month. Notably, transitioning to PDS I sutures from PDS II has mitigated knot irritation, further optimizing patient outcomes with minimal motion restriction or discomfort.

## Disclaimers:

Funding: No funding was received under any form for this study.

Conflicts of interest: The authors, their immediate families, and any research foundations with which they are affiliated have not received any financial payments or other benefits from any commercial entity related to the subject of this article.
